# Investigation of TGFBI (Transforming growth factor beta-induced) Gene Mutations in Families with Granular Corneal Dystrophy Type 1 in the Konya Region

**DOI:** 10.4274/tjo.galenos.2019.55770

**Published:** 2020-04-29

**Authors:** Fatma Malkondu, Hilal Arıkoğlu, Dudu Erkoç Kaya, Banu Bozkurt, Fehmi Özkan

**Affiliations:** 1 Selçuk University Faculty of Medicine, Department of Medical Biology, Konya, Turkey; 2 Selçuk University Faculty of Medicine, Department of Ophtalmology, Konya, Turkey; 3 Konya Numune Hospital, Clinic of Ophtalmology, Konya, Turkey

**Keywords:** Granular corneal dystrophy type 1, TGFBI gene, R555W mutation

## Abstract

**Objectives:**

Granular corneal dystrophies (GCD) are characterized by small, discrete, sharp-edged, grayish-white opacities in the corneal stroma. Among the genes responsible for the development of GCD, the most strongly related gene is transforming growth factor beta-induced (*TGFBI*), located in the 5q31.1 locus. Studies show that R124H in exon 4 and R555W in exon 12 are hot-spot mutations in the *TGFBI* gene that lead to GCD development. In this study, we aimed to investigate these two hot-spot mutations in exons 4 and 12 of the *TGFBI* gene and other possible mutations in the same regions, which code important functional regions of the protein, in Turkish families with GCD1 and to determine the relationship between the mutations and disease and related phenotypes.

**Materials and Methods:**

The study included, 16 individuals diagnosed with GCD type 1 (GCD1), 11 of these patients’ healthy relatives, and 28 unrelated healthy individuals. DNA was obtained from peripheral blood samples taken from each individual and polymerase chain reaction was used to amplify target gene regions. Genotyping studies were done by sequence analysis.

**Results:**

The R124S mutation in exon 4 of *TGFBI* was not detected in the patients or healthy individuals in our study. However, all individuals diagnosed as having GCD1 were found to be heterozygous carriers of the R555W mutation in exon 12 of *TGFBI*. This mutation was not detected in healthy family members or control individuals unrelated to these families. In addition, we detected the silent mutation F540F in exon 12 and c.32924 G>A substitution in an intronic region of the gene in a few patients and healthy individuals.

**Conclusion:**

Our study strongly supports the association of GCD1 with R555W mutation in exon 12 region of the *TGFBI* gene, as reported in the literature.

## Introduction

Corneal dystrophy is a group of progressive, often bilateral, usually inherited diseases characterized by non-inflammatory opacification.^1^ The diagnosis and classification of corneal dystrophies is based on corneal findings observed on slit-lamp biomicroscopic examination. Classification is based on clinical, pathological, and genetic features (IC3D) (Table 1).^2^ According to this classification, 5q31-linked corneal dystrophies are often caused by mutations occurring in the transforming growth factor β-induced (*TGFBI*) gene.^1^ Different mutations in *TGFBI*, which is located in the *5q31* gene locus, lead to granular corneal dystrophy types 1 and 2 (GCD1 and 2), Reis-Bückler corneal dystrophy, lattice corneal dystrophy (LCD), and Thiel-Behnke corneal dystrophy.^3^ GCD1, a member of the corneal stromal dystrophy group, is a slow-progressing, mostly asymptomatic autosomal dominant dystrophy that usually appears in childhood. The corneal opacities are small, discrete, sharp-edged, gray-white, and located in the central stroma, while the peripheral cornea is clear. Although there is no visual impairment in the early stage, the deposits on the stroma layer merge and reduce vision as the disease progresses.^4^

The *TGFBI* protein encoded by the *TGFBI* gene is produced mainly by the epithelium and partly by stromal fibroblasts called keratocytes, and is transported to the stroma.^5^^,6^ The stroma, which comprises about 90% of the cornea, has a rich matrix and special importance both structurally and functionally. The lamellar structure, arranged in a hexagonal pattern of type I and type V heterotrimeric collagen fibrils in the stroma matrix, is essential for the cornea to remain transparent.^7^ Along with collagen types VI, XII, and XIV, the proteoglycans decorin, lumican, keratocan, mimecan, biglycan, and fibromodulin provide structural support for the assembly and positioning of collagen fibrils.^8^ The *TGFBI* protein, a matricellular protein, establishes and maintains matrix organization by binding to integrin via FAS1 domains containing the arginine-glycine-aspartic acid (RGD) sequence, the integrin-binding motif in matrix elements. The *TGFBI* protein includes a signal peptide sequence (Met1-Ala23), cysteine-rich EMI domain (Gly45-Ala99), 4 FAS1 domains (Ala100-Pro635) containing 140 amino acids each, and the RGD motif at the C-terminal.^9^^,10^

To date, more than 50 mutations identified in the *TGFBI* gene have been associated with corneal dystrophies and these mutations have mostly been identified in the codon Arg124 in the first FAS1 domain or in the codon Arg555 in the fourth FAS1 domain.^11^ GCD1 has most frequently been associated with a missense mutation in the CG dinucleotide in codon 555 of *TGFBI* that causes the substitution of arginine (Arg/R) to tryptophan (Trp/W).^12^ In this study, individuals diagnosed with GCD1 and their families were evaluated using DNA sequence analysis for the presence of the two hot-spot mutations mentioned in the literature and other mutations in the exon 4 and exon 12 regions, which encode the functionally important first and fourth FAS1 domains of the *TGFBI* protein, respectively.

## Materials and Methods

### Patient and Control Groups

**Figure 1:**
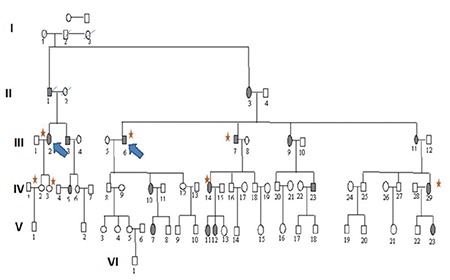
Family tree of family 1. Shaded symbols indicate individuals diagnosed with granular corneal dystrophy type 1 (GCD1), the arrow indicates the proband, and members included in the study are indicated with stars

**Figure 2:**
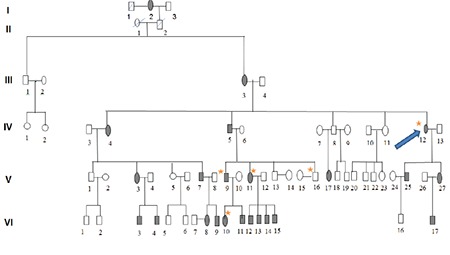
Family tree of family 2. Shaded symbols indicate individuals diagnosed with granular corneal dystrophy type 1 (GCD1), the arrow indicates the proband, and members included in the study are indicated with stars

**Figure 3:**
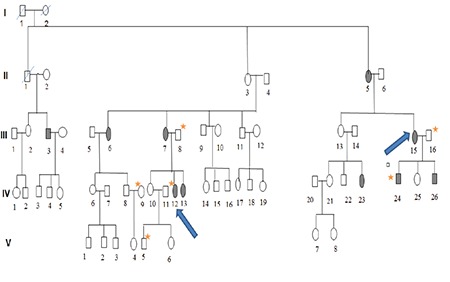
Family tree of family 3. Shaded symbols indicate individuals diagnosed with granular corneal dystrophy type 1 (GCD1), the arrow indicates the proband, and members included in the study are indicated with stars

**Figure 4:**
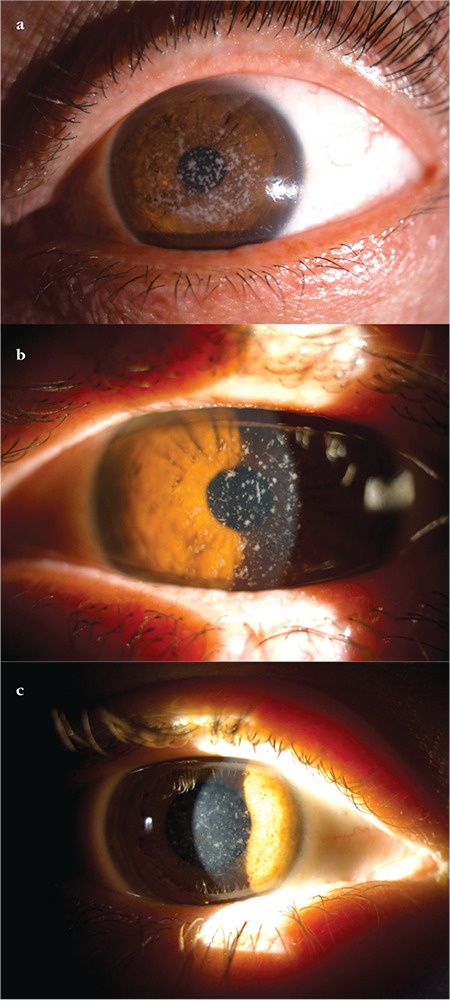
Anterior segment photographs of a) a 72-year-old male patient (III-6) from family 1 who had photophobia and vision loss, b) a 73-year-old female patient (IV-12) from family 2, and c) a 20-year-old female patient (IV-12) from family 3. Small, discrete, sharp-edged, breadcrumb-like grayish-white opacities are observed

Approval for the study was obtained from the Selçuk University Faculty of Medicine Ethics Committee (2017/193). All subjects were informed and their consent was obtained prior to their participation in the study. Three individuals who presented to and were diagnosed with GCD1 in the ophthalmology department of Selçuk University Faculty of Medicine and their families were included in the study. Four-generation family trees of these 3 GCD1 patients were created (Figures 1, 2, 3). The history of each patient with regard to age, signs and symptoms, disease progression, genetic diseases, and medications used was recorded. Relatives of the patients underwent complete ophthalmological examination with assessment of uncorrected and corrected visual acuity. In slit-lamp biomicroscopic examination, the density and location of corneal deposits were examined and anterior segment photographs were taken (Figure 4). The properties and depths of the deposits were evaluated on anterior segment optical coherence tomography (OCT) and a detailed *in vivo* examination of the cornea at the cellular level was performed with confocal microscopy. Sixteen of the evaluated family members were diagnosed with GCD1, while no disease was detected in 11. In addition, 28 individuals who were unrelated to these families or with one another and had normal ophthalmic examination findings were included in the study as the control group. Peripheral blood samples of 6 cc were collected from each participant into EDTA tubes for DNA extraction.

### Determination of Target Gene Regions and Primer Design

The nucleotide sequences of the *TGFBI* gene were obtained from the National Center for Biotechnology Information (NCBI)  GenBank™ with access number NM_000358. We planned to use primers designed to include the entirety of the exon 4 and exon 12 regions of the TGFBI gene, shown in the literature to be particularly associated with the disease, and therefore we used appropriate primers reported in the literature (Table 2).

### Polymerase Chain Reaction (PCR) Procedure

Gradient PCR was used to determine the appropriate melting temperature for the 2 pairs of primers to be used in the study. Samples were amplified using the temperatures determined for each primer pair in gradient PCR. PCR was conducted in 30-µL reaction volumes containing 1X PCR buffer, 0.4 mM primer, 0.6 mM deoxyribonucleotide triphosphate, 0.1 unit Taq polymerase, and 100 ng DNA. PCR conditions were as follows: initial denaturation at 94 °C for 5 min, followed by 35 cycles of denaturation at 94 °C for 30 s, primer annealing for 45 s at the temperature determined by gradient PCR for each primer, and elongation at 72 °C for 45 s, with a final elongation at 72 °C for 2 min. PCR results were evaluated in 1% agarose gel electrophoresis and photographed.

### DNA Sequence Analysis

Sequence analysis was performed on the target DNA regions amplified with PCR. The sequences were visualized as chromatograms in the FinchTV software and matched with sequences in the ) GenBank™ using the NCBI BLAST program from (https://www.ncbi.nlm.nih.gov/gene). The BLAST results and chromatograms were carefully compared to evaluate changes in the sequences. Two-way sequence analysis was performed to prevent potential errors in DNA sequence analysis. Thus, mutations were identified in both directions for a highly reliable evaluation.

## Results

**Figure 5:**
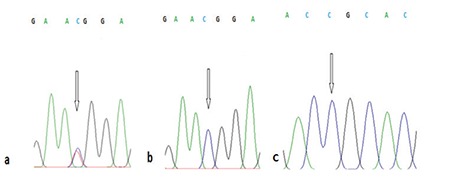
Sequence analysis results for the 1663C→T (Arg555Trp) change in the exon 12 region. a) Electropherogram of C/T heterozygous genotype, b) Electropherogram of C/C homozygous genotype. No individuals with the T/T homozygous genotype were detected in this study. c) Sequence analysis results for 370C→T (Arg124Ser) in the exon 4 region. All individuals included in the study were C/C homozygotes

**Figure 6:**
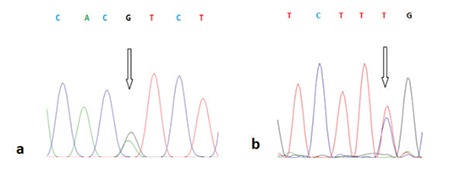
Electropherogram of the c.32924G→A (rs2072239) heterozygous genotype detected in the intron 12 region, b) Electropherogram of the 1620T→C (Phe540Phe) heterozygous genotype in the Exon 12 region

According to sequence analysis results for the exon 12 region of the *TGFBI* gene, all patients diagnosed with GCD1 were heterozygous carriers of the hot-spot Arg555Trp (c.1663C>T) mutation (Figures 5a). This mutation was not detected in healthy family members or the control subjects (Figure 5b). In addition, the single nucleotide polymorphism (SNP) Phe540Phe, which is a silent mutation previously reported in the GenBank™, was detected in the exon 12 region in sequence analyses of 4 GCD1 patients and 1 unaffected participant (IV-14 and IV-12 from family 1; V-12 and V-16 from family 2; and in 1 unrelated control subject) (Figure 6a and b). A SNP (rs2072239) located in the intronic region of the target gene region was detected in 3 affected family members (IV-14 from family 1, V-12 and VI-12 from family 2), and 2 unaffected family members (V-11 from family 2 and IV-25 from family 3).

The Arg124Ser mutation was not detected in the exon 4 region of *TGFBI* gene in any of the affected or unaffected participants. No other mutation or polymorphism was detected in the exon 4 region (Figure 5c).

## Discussion

The *TGFBI* gene has a key role in the genetic basis of GCD1 and encodes the *TGFBI* protein, which is synthesized primarily by epithelial cells and partly by keratocytes in the cornea, and released into the stromal matrix. This protein plays an important role in establishing and maintaining matrix organization, and heterogeneity in the *TGFBI* gene, encoding this protein, leads to different clinical presentations. In particular, mutations at R124 and R555 in the exon 4 and exon 12 regions, which encode the first and fourth FAS1 domains of the protein respectively, have been shown to cause different types of dystrophies. Of the mutations that occur at position 124 of exon 4, the R124H mutation results in GCD2 (granular-lattice, Avellino), the R124C mutation results in LCD type 1, and the R124L mutation results in Reis-Bückler corneal dystrophy. Of the mutations at position 555 in exon 12, the R555W mutation causes GCD1 and the R555Q mutation causes Thiel-Behnke corneal dystrophy.^3,11^ Different mutations in the *TGFBI* gene, even if they occur at the same position, have been shown to cause different corneal dystrophies by altering folding, proteolysis, accumulation, and half-life (turnover) of the protein.^15,16,17,18^

*TGFBI* and Drosophila fascilin I are highly homologous members of a superfamily of proteins containing FAS1 domains.^14^ In a study using a homology model, it was reported that mutations in codons Arg124 and Arg555 can alter stability by affecting protein-protein interactions. The Arg124 residue is located between the α1 and α2 helixes of the first FAS1 domain, while the Arg555 residue is located in the binding region of the α3 and α4 helixes of the fourth FAS1 domain, and both are predicted regions of proteolytic degradation.^10^ It was demonstrated by NMR (liquid-state nuclear magnetic resonance) spectroscopy that proteolysis occured between the Arg557 and 558 residues of the normal *TGFBI* protein, but with the Arg555Trp mutation this domain became resistant to proteolysis.^19^

Mutations occurring at different sites of the *TGFBI* gene result in the accumulation of *TGFBI* protein as insoluble residues of varying forms in different layers of the cornea, thereby causing clinical variations. More than 50 mutations detected in the *TGFBI* gene have been associated with various corneal dystrophies characterized by the extracellular accumulation of insoluble mutant protein (amyloid, hyaline) in the cornea, such as granular, lattice, Avellino, Bowman layer types 1 and 2, and basal membrane corneal dystrophy.^12^ FAS1 domains are regions with a compact globular structure containing an α-helix and α-layer.^20^ Two hot-spot mutations at positions R124 and R555 in the first and fourth FAS1 domains of *TGFBI*, respectively, are the most frequent mutations in different ethnic populations.^21^ The mutation most commonly associated with GCD1 is the Arg555Trp mutation, which occurs in the fourth FAS1 domain. According to the IC3D classification, this mutation is accepted as the mutation associated with GCD1. Although rare, the Arg124Ser mutation in the first FAS1 domain was also shown to be associated with GCD1.^22^

In one of two related studies conducted in Turkey, Kiratlı et al.^23^ screened the exon 4 and 12 regions using the single-strand conformation polymorphism (SSCP) method and identified the Arg555Trp mutation in a large Turkish family with 52 members, 26 of whom had GCD1. Yaylacioglu Tuncay et al.^13^ obtained the same result in 12 GCD1 patients analyzed for both hot-spot mutations. Although the exact prevalence of corneal dystrophies in Turkey is unknown, there are a large number of affected families, especially in the Central Anatolian region.

In this study, the Arg555Trp mutation was detected in 16 individuals with GCD1 who belonged to 3 large families from the province of Konya and its surroundings. It has been shown that while the Arg555 residue in a normal *TGFBI* protein is exposed and subject to lysis, the Trp555 mutant residue is buried in the hydrophobic cavity of the fourth FAS1 domain of the protein.^19^ It was also demonstrated in a molecular dynamics simulation that this mutation causes the C-terminus of the α3 helix of the lytic region of this mutation to be less flexible, thereby conferring proteolytic resistance.^19^ These findings explain the accumulation of deposits of *TGFBI* in the matrix in the pathogenesis of GCD1. The degradation of extracellular matrix proteins is just as essential as their production for cellular processes such as tissue development, remodeling, and repair, and the disruption of this balance plays a role in the pathogenesis of many diseases, including corneal dystrophies.

Our findings in this study support the view expressed in the literature that the R555W mutation in the *TGFBI* gene is involved in the genetic basis of GCD1. In this study using sequence analysis to evaluate the entirety of the exon 4 and exon 12 regions, which encode the functionally critical FAS1 domains, we detected two variations in the exon 12 region other than the known hot-spot mutation at position R555. One of these was a variation at position 540 that was a silent mutation causing no amino acid change. No relationship was observed between GCD1 and the Phe540Phe mutation that we detected in 3 GCD1 patients and 2 unaffected individuals. There are studies in the literature suggesting that a missense mutation (Phe540Ser) and a deletion (Phe540del) at the same position are associated with LCD1/3A.^24,25^ It is suggested that the mutation we detected in this position, which is considered a possible hot-spot, is not associated with the disease because it did not cause changes in protein structure and was also detected in unaffected individuals. In this study, no relationship was established between the GCD1 phenotype and mutation rs2072239, an intronic SNP that we identified in 3 GCD1 patients and 2 unaffected individuals.

Performing genetic analyses in the families of affected individuals is important for diagnosis in the early stages or before the appearance of clinical symptoms and also for clinical follow-up and treatment planning. Genetic analyses are also used to differentiate the different corneal stromal dystrophies that have similar clinical features. In GCD2, which is caused by the R124H mutation and is most often clinically confused with GCD1, the granular deposits are fewer in number and lattice-like linear and stellate deposits are observed in later stages.^26^ In light microscopy studies evaluating histopathological structures, Masson trichrome-positive deposits are observed in GCD1, while GCD2 exhibits hyaline and amyloid deposits that stain with Masson trichrome and Congo red, found from the basal epithelium to the deep stroma.^26^ On the other hand, genetic analyses are important for definitive diagnosis of individuals affected by Reis-Bückler and Thiel-Behnke corneal dystrophies, which are difficult to diagnose based on clinical and histopathological features.

Another point to consider is that in *TGFBI*-related dystrophies, clinical differences can be observed even among members of the same family. This supports the idea that other matrix elements may also contribute to the effect of *TGFBI* protein in the formation of corneal deposits. Other factors independent of *TGFBI* may be involved, or there may be factors that regulate *TGFBI* or epigenetic factors. It has been shown in vitro and in vivo that Sp1 and Sp3 binding sites in the promoter region of the *TGFBI* gene are occupied by these factors in cells expressing TFGBI. Regarding epigenetic mechanisms, it was shown that H3K4Me1,3 is enriched and H3K27Me3 is suppressed in the promoters of *TGFBI* and matrix-related genes.^27^ However, more epigenetic studies are needed.

GCD1 is more common than other corneal dystrophies due to its autosomal dominant inheritance, and although the increase in deposits with age impairs vision and eventually necessitates corneal transplantation, the deposits recur in the graft and current treatment approaches do not provide permanent recovery; therefore, genetic disorder-regulating gene therapy trials are needed for this disease. Clarifying the structure, function, and regulatory processes of the *TGFBI* gene and protein and understanding the genetic architecture of different corneal dystrophies and at what stages the molecular mechanisms are disrupted will determine future treatment strategies.

## Figures and Tables

**Table 1:**
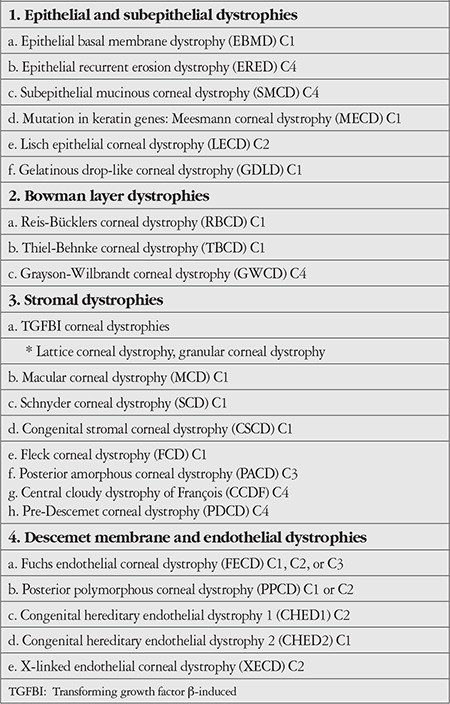
Classification of corneal dystrophies^2^

**Table 2:**
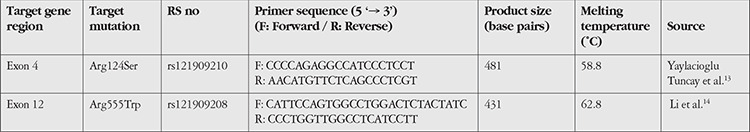
Hot-spot mutations in the exon 4 and exon 12 regions of the *TGFBI* gene and primer sequences used to amplify these regions
